# Appreciation of Art as a Perception *Sui Generis*: Introducing Richir’s Concept of “Perceptive” *Phantasia*

**DOI:** 10.3389/fpsyg.2021.576608

**Published:** 2021-05-28

**Authors:** Dominic Ekweariri

**Affiliations:** ^1^Department of Philosophy, Institut für Transzendentalphilosophie und Phänomenologie, Bergische Universität Wuppertal, Wuppertal, Germany; ^2^Marc-Richir-Archiv, Bergische Universität Wuppertal, Wuppertal, Germany

**Keywords:** perception in the visual system, Heidegger’s everydayness, “perceptive” *phantasia*, “Sache” and virtuality, *Leiblichkeit*, artworks, Richir, truth being and world

## Abstract

In the *Origin of the work of art*, Heidegger claimed that the work of art opens to us the *truth of Being*, the opening of the world. Two problematics arise from this. First, his idea of “world-disclosure” evoked a sense of *everydayness* (which captures, for me, the idea of credulism in perception). Second, the senses of *truth*, *Being*, and *world* are metaphysically condensed. Hence the question: how then could the “truth of Being” or the “world” that artworks reveal be experienced? Among other ways (*mimesis*, imagination, perception, etc.) by which artworks are experienced, I choose to examine perception since it confirms this idea of *everydayness*. The questions that confront us to this effect are: can perception lead us into, to encounter, this world opened by artworks? Does the nervous/visual system suffice to enter into that world in which the artist invites us? This is where Richir becomes important. In response to the first problem, he shows that the “perception” (experience) of artworks is beyond mere *everydayness* since artworks open for us a world that “never was” and “never will be” (i.e., “virtuality” and not a veridical sense of *everydayness* as captured in the perceptive act that is object-related). This is because the material stuff or object given in perception is neutralized by the *phantasia* to become what Richir calls *Sache*. This *Sache* is in itself a phenomenon that is disclosed in artworks. In response to the second problem, Richir shows how artworks cannot disclose just metaphysical categories of *Being, truth, or world*. The disclosure has to be phenomenological, corporeal, and affective. He therefore proposes another mode of “perception” beyond *mere perception* in a revolutionary interpretation of the husserlian “perceptive” *phantasia.* With this, he shows how the aforementioned metaphysical condensations are liveable in experience. I concretize this with an illustration from the theater. Finally, I suggest participation as a phenomenological approach that can make both Heidegger’s and Richir’s intuitions meaningful.

## Introduction

To the question, “what is at work in the work (*sic*: of art)?”, Heidegger responded: “in the work is the event of *truth* at work, if here the *opening of entities* occur in that which and how it is. In the work of art, *truth* of entity has come into play [….] The work of art *opens up the Being of entities* in its own way” (Heidegger, 2012, my translation). Though Heidegger’s primary concern was not the *tool or entities*, but the nature of the *works of art* (*Kunstwerke*), it was the contemplation of the latter that manifested the former, i.e., in its *Being*, because it could speak to the contemplator. This idea is interesting if we consider that Heidegger’s effort to investigate the determination of *the thing* (*Ding*) in its *thingliness* (extended to all entities: tools, things, and works) via the three popular western interpretations of the thing did not yield positive results. None of the three interpretations of the thing succeeded in exposing that which is at work in the work of art. Rather, they among other things prevented the way to the *artness* of artworks, and in so doing blocked the way to the reflection of Being (Heidegger, 2012). According to Tonner, the three determinations of *the thing* blocked out the way to Being “because in each case the thing is defined in terms of its relationship to lived experience or to its possible relationship with a subject” (Tonner, 2014). This point is important because it underlines Heidegger’s motivation. This inability of the traditional determination of things to reveal artness of artworks results from the fact that in them there was an anticipation of direct experience of entities. So the “anticipation prevents reflection of Being of the respective entities” (Heidegger, 2012). It is therefore clear that Heidegger made a turning point away from this sort of subjective familiarity with entities and art. He understands art, as Plumpe (1993) says, no longer from the position of subjectivity, but rather from an ontological standpoint of Being as a way in which truth happens as an event. Rather than subjectivity, the metaphysical and ontological categories of Being, Truth and World, are more crucial. Thus, we ask: how are these metaphysical categories then given to us in the experience of artworks?

The opening of an entity, in its Being, appears, in Heidegger, as another way of expressing the happening of *truth* (Heidegger, 2012). But not only do artworks open the way to Being; in them too is *truth* at work as an event---a coupling that appeared in *Being and Time*^1^. Before I explore the implications of this event or the happening of truth, I deem it necessary to point out that the *happening of the truth*^2^ appears to be interchangeable with the *opening of the world*. A passage where Heidegger asked and responded might help: “But how does truth happen? We answer: it happens in opening of Being… Opening of little essential ways. One of these ways, how truth happens is the artness of artworks. *In setting up a world* and producing earth, *the work of art* is the contestation of that conflict in which the unconcealedness (*Unverborgenheit*) of entity in its totality, i.e., *the truth* is secured” (Heidegger, 2012). In other words, the *setting up of a world* is one way in which *truth* or the unconcealment of the entities happens.

If *truth* and *world* correspond as we have seen, then we might ask, how can this world be understood? In Young’s reading, truth implies a “horizon of disclosure” (Young, 2001). It is within this *horizon of disclosure* in which everyone finds (ontology) himself that Heidegger understands as world. Thus, Young writes in this purview: “In sum, then, ‘world’ is the background, and usually unnoticed understanding which determines for the members of an historical culture what, for them, fundamentally, there *is*” (Young, 2001).

If “world” documents this ontological structure, and if truth is to be understood in this ontological context, then the work of art must, according to Heidegger, express and reveal this fundamental ontological structure. The said work of art has to open up world-defining events, e.g., a Greek temple, the Bamberg Cathedral, or the *Igbo ukwu*, and unconceal not only the beings in those worlds but also the worlds of people living in that time. In the same way, it can lay bare the significance of an individual’s space just like the shoe paintings by Van Gogh disclosed the peasant woman’s quotidian world (everydayness). In this last instance, we have before us an artwork painted by Van Gogh: the shoes of the peasant woman could reveal the space in which she belongs; the shoes are what they are in the familiar ground of the farm. Contemplating the painting of Van Gogh, that *world* of everydayness of the peasant woman is unconcealed: the travail of work stages, the toughness of the slow gait through the largely stretched furrow, the solitude of the farm track pushing under the soles during the setting evening, the silent shouts of the earth, when it silently brings forth fruits of the earth, and when it inexplicably fails to bring forth fruit in the barren wasteland of the winter field. In it one sees the uncomplaining trepidation for the assurance of bread, the non-verbal joy of the survival of hardship (Heidegger, 2012). What happens when the shoes become a piece in museum collection? In that case, the world which they open for us would have collapsed no matter how they were preserved and optimized for the art industry. The *truth* of their *Being* would have been forever lost. In other words, works of great arts, to which Heidegger was particularly referring, lose their essence when not rooted in ontology. It is in these last instances that the categories of *truth of Being* and the *world* could be understood.

It is obvious that Heidegger is defending a radical ontological approach to works of art in contrast to two attitudes: the first is *a heavy aesthetization*^3^ of artworks, in which Dasein is first an object-ignorant subject in order later to encounter a subject-ignorant object and the second is *a Platonian ontological critique*^4^ that beauty was not to be found in the object of art but derived from the world of forms. Heidegger’s ontological approach posits Dasein *is always standing outside of itself; this Dasein is immersed in the world of everydayness*. In George Steiner’s words: “To be at all is to be worldly” (Steiner, 1987:85). It is this ontological relationship to the background world of everydayness in which humans are immersed that artworks should evoke. But this world-disclosure evokes for us a sense of everydayness. In Heidegger’s “Being and Time,” everydayness (*Alltäglichkeit*) captures a conventional, “routine” or “habitual,” “less-than- ideal,” inauthentic, and “unreflected” way of being-in-the-world, as Thompson noted (Thompson, 1983). This captures for me the idea of an inauthentic and unreflected approach to perception, a sense of credulism in the veridical impression of reality, which can give the impression that *pure perception* could suffice to disclose to me the essence of artworks. I name this the *first problem*. Thus, we are motivated to ask: does perception suffice to disclose the essence of art to us? In other words, are the eyes or the nervous systems, by their registration of signals from the world, sufficient to open to us that world which artworks articulate? If they are not, what must happen to complete the insufficiency of mere perception via the nervous systems?

If we followed Heidegger’s thought, we would be lost as to how this world of *everydayness*, the truth of Being in artworks, can be given to us in concrete experience. In *Poetry, Language, Thought*, Heidegger suggests an approach, which is purely phenomenological (Heidegger, 1971). But he was unable to show us how these condensed metaphysical categories—of truth of Being and the world—could be given to us in experience. I name this the *second problem*.

According to Richir, the phenomenological is strictly required by the aporias of the metaphysical (Richir, 1975). My motivation in this paper is simply to overcome the *first* and *second* problems by highlighting how the phenomenology of Richir might overcome the puzzles of metaphysics presented in Heidegger and stated above. Through the revolutionizing interpretation of the husserlian “*perceptive” phantasia*, Richir shows us, in opposition to an unreflected approach to *pure perception*, how to encounter the metaphysical *truth of Being* (given phenomenologically as *Sache*, “the real,” “the sensible” (*sens*) and involving affectivity and corporeality) that every work of art bears. In so doing, he makes Heidegger’s intuitions fruitful, thus solving the *second problem*. Richir also shows how the *world* disclosed to us by the works of arts “has never been” and “will never be” (thus beyond the credulism of object-related perceptive acts mapping out everydayness) since the material stuff and objects are neutralized and since the world that is disclosed is essentially “virtual,” solving the *first problem*.

To achieve this goal, the paper is divided into three main parts: The *first part* is dedicated to the question of whether perception via the nervous systems is sufficient to capture the essence of art. The background I give is that of Plato who, in the *Phaedrus*, tried to articulate the experience of the metaphysical within the setting of visual perception^5^. The eyes occupied therein a central position. But modern science has extended the pathway of perception to include the nervous system, including the brain. The analyses of some research results from neuroscience could give the impression that we could understand the contemplation of artworks only in terms of object perception via the nervous system. Against this backdrop, I argue in line with Richir that the physical constitution of optical signals is insufficient for mere perception and *a fortiori* for the contemplation of the essence of artworks. In the *second part*, I shall argue with Richir that what is disclosed in the work of art is “perceived” no longer in terms of object-relatedness but as *Sache*, in the transitional space of free play and including a figuring and infigurable part. The argument culminates in showing that that which is “perceived” in the work of art is “perceived” as “Sache,” in the “perceptive” *phantasia*, and is astride a figuration and infiguration, in transition between “reality” and *phantasia*, but illocalizable in perceptive and imaginative objects as such, i.e., not as real objects and not as phantoms. After showing that the *Sache* is in the pole neither of material reality nor irreality, I shall show in the *third and last part* that the *Sache* is purely virtual. Contemplating a piece of work implies perceiving “something” that is *in presence* but *not present*, whose presence is virtual and whose effect is real (“First problem” is solved). I shall then proceed to show that while lying in the sphere of indetermination, the *Sache* mirrors essentially the transgressing of the metaphysical condensations, when these are given as codifications in socio-cultural and historical milieus, etc. In this sense, the *Sache* is captured by those elusive, dissatisfactory feelings that artworks try to communicate, making the essence of art somewhat elusive and reduced to the barest minimum—where the barest minimum highlights the aspect of phenomenality. In *this barest minimum, truth* or *Being* is phenomenologically translated as appearance, thereby putting Metaphysics into parenthesis (“Second problem” is solved). The last idea is effectuated by a process of phenomenalization where the kinesthesis of *Leiblichkeit* (corporeality) and the *phantasia-affection* set up the formation of phenomenon, a sort of inchoate and indeterminate sensibility. This sensibility is only affectively accessible via a special type of “perception” that completes the “perception” of the “perceptive” *phantasia: an active, non-specular mimesis from without*. I concretize this with an illustration from the theater. Finally, I suggest participation as a phenomenological approach that can make both Heidegger’s and Richir’s intuitions meaningful.

## Perception

Perhaps the best way to begin a discussion of perception of artworks would be by pointing out that it was Plato who first in the *Phaedrus* documented the role of the eyes in perceptive or visual experience. Though Plato never made mention of the work of art here—art was in that epoch indistinguishable from other crafts since it was, like every other work of an artisan, the result of *techne*—the thematization of beauty is with reference to the object of beauty, for which we understand objects or works of art. A chapter in the *Phaedrus* entitled “The Vision of Beauty” understood beauty in terms of a visual experience and treated it as a brilliance. An excerpt will do: “Beauty it was ours to see in all its *brightness* in those days when, amidst that happy company, we *beheld with our eyes* that *blessed vision*, ourselves in the train of Zeus […] steadfast and blissful were the spectacles on which we gazed” (PLATO, 1952: 250b-c, my italics). The senses perceive this brilliance in the works of art. The eyes are of particular interest. But if the eyes enjoy this prerogative^6^, it is because “sight is the keenest mode of perception vouchsafed us through the body” (PLATO, 1952: 250d). The consequence is that without this essential aspect, nothing is perceptible. Without the mediation of this keenest of all the senses, forms such as temperance and justice (the moral forms) would be invincible. In contrast, this imperceptibility did not apply to *beauty* since *it* was lived through the medium of the sense of vision (PLATO, 1952:250b). It is therefore not surprising that for thinkers all through history, the eyes occupy a central position in the experience of artworks. Merleau-Ponty cited an instance of this: “The eye … through which the beauty of the universe is revealed to our contemplation is of such excellence that whoever should resign himself to losing it would deprive himself of the knowledge of all the works of nature, the sight of which makes the soul live happily in its body’s prison, thanks to the eyes which show him the infinite variety of creation…” (Toadvine and Lawlor, 2007). The above Platonian intuition has been expanded to include not only the eyes but also the entire visual circuit that comprises the nervous systems in aesthetic experience. It is therefore of utmost importance to us to inquire to what extent the visual circuit is sufficient to experience the essence of artworks and the world they open to us. Before then, we would cursorily glance through a few of the recent studies—both by artists and by neuroscientists—in the perception of art.

Here we find the puzzling revelation of how we come to perceive (recognize) that artwork before us. There is no better description than a neurobiological approach: it begins with the traveling of light from the surrounding world to our retina, with the lenses focusing the light rays that travel across it in order to adjust the position of boundaries and intensities from the surroundings. The information passed on via retina stimulation is to be processed; there are some advanced computational theories (neural models^7^) on how the information regarding motion, color, and orientation is deposited in the brain (but how the brain arrives at perceptual recognition happens in a later stage). We call this the “early stage” following Robert Pepperell’s reference to experts of visual perception. At this stage, as he said, the viewer is only able to see “forms, lines, colors, motions, etc.” What s/he lacks at this stage is recognition; the perception has no “specific meaning.” For this impasse to be overcome, another cognitive activity—and this is the second stage—has to conclude the process by supplying the primitive elements of forms, lines, and shapes (which are processed in the retinae and in the cortex, Pepperell, 2019) with what Pepperell called “semantic information.” This means that the shapes and forms are imposed with meanings arising from concepts, memories, or previous knowledge. This is more advanced work our visual system has to perform to be able to build perceptible images from primitive elements like edges, corners, contrasts, colors, *etc*. Without this “higher level” stage, the recognition of what is given in a work of art would be impossible. Following Martha Farah, Pepperell cites an instance of “visual agnosia”—indicating a neurological condition in which the eyes and brain of the person can register a stimulus without assigning meaning to it—which confirms how these two stages are separated from each other, but how their collaboration is necessary for vision (Pepperell, 2012). In other words, the first can continue to function without the second.

If the above scientific description is true, then it gives us a clue of two ways of experiencing the works of art. The first derives from visual agnosticism. A visual agnostic victim has documented this visual experience; he saw the “richly elaborated but formless visual ‘stuff’ that lacks specific recognizable objects” (Pepperell, 2012). The second would be the very opposite: a state of recognition with a determinate object perception. In an article titled *What does the brain tell us about abstract art?*, Vered Aviv has explored these two aspects in what he called “the two ends of a continuum between *representational art* and *abstract art*” (Aviv, 2014). The two aspects reveal the determinacy and indeterminacy of artworks. In the *Neural Correlates of Beauty*, Kawabata and Zeki’s result, using the functional MRI to address the question of specific brain areas—correlates—that were mobilized during the viewing of beautiful paintings, revealed that “the perception of different categories of painting” (e.g., portrait, landscape, still life, or abstract composition; thus representational and abstract art) “are associated with distinct and specialized visual areas of the brain” (2003:1699). The several categories of paintings indicated activity in specific areas of the brain (like the lateral and middle occipital gyri, in the middle of fusiform gyrus implicated in face-recognition and amygdala, in the medial orbito-frontal cortex, the anterior cingulate, the motor cortex, etc.). However, for abstract art, “at the corrected level, no activity was produced by the CA (*sic:* cognitive conjunction approach)” (1700). Consequently, the researchers concluded that for all the categories of painting (representational art) there was maintained a relative linear increase of activity during the viewing of beautiful stimuli. This was not the case during the viewing of ugly stimuli and abstract paintings (Kawabata and Zeki, 2004). A similar result can be found in Lengger et al. (2007) for whom representational artworks evoked more associations and an increased activation in certain brain areas due to object recognition. Abstract art failed to activate a specific brain region. In reference to Cupchik et al. (1992) and Augustin et al. (2008, 2011) Vered Avid concluded that abstract art was processed through the brain’s analytic path of style and had little to do with pictorial content. The stylistic processing of abstract artworks required more time as there was a very high probability that viewers of artworks may not have seen these before. In relation to this stylistic processing of abstract art, Pepperell wrote, “indeterminate images resist immediate classification” (2019: 117). In contrast, processing of pictorial content was always fast since viewers did not need much time to categorize the items they viewed, as they must have internalized these over time (Aviv, 2014). Aviv pointed out, in reference (Pihko et al., 2011) to the works of Taylor et al. (2011) who had investigated eye tracking of viewers of representational and abstract arts, that the viewers’ eyes for representational art “tend to gaze mostly on salient features in the painting (e.g., eyes, nose, trees, signature, etc.),” whereas for abstract art they “tend to scan rather uniformly the surface of the whole canvas” (Aviv, 2014); these studies go a long way to confirm that while the perception of abstract art might be characterized by indeterminacy of the objects disclosed, in representational art what we have is the opposite: the determinacy of the objects perceived.

Given the above neuroscientific findings, we might begin to think that in abstract art, works of art are not object-specific, and that they give the eyes and neural paths the capacity of forming free association, whereas representational artworks are object-related, limiting the eyes to be attentive to objects. The consequence would be that perception is not flexible in the case of representational art. Yet some neurobiological studies have indicated the ostensible compulsion by humans to impose objects even in objectless abstract arts. In one such study, Pepperell, in collaboration with scientists at the Max Planck Institute for Biological Cybernetics and at the University of Zürich, made a perplexing discovery: test subjects reported seeing specific objects in art images that lacked these objects. He wrote that despite his numerous efforts to remove traces of identifiable forms, “on average subjects reported familiar objects up to 36% of the time (in some paintings objects were seen 52% of the time).” As if that were not enough, subjects tended to see objects 18% of the time in what can be understood as “a set of entirely abstract painting.” The study concludes: “Our findings indicate that this seemingly effortless process (of recognition) occurs not only with familiar objects, but also with indeterminate stimuli that do not contain real objects” (Pepperell, 2012). The consequence leads to the hypothesis that the primate’s brain “is a compulsory object viewer”; that is, it tends strongly to organize indeterminate objects into recognizable and “coherent images” (Pepperell, 2012). Between the indeterminacy and the determinacy of artworks, the nervous system (i.e., the eyes and the brain, etc.) works hard to overcome this cluelessness by a compulsive, automatic imposition of images.

What phenomenological consequences would this imply for the experience of the works of art via perception? That the nervous system (for instance, the eyes as visual organ or the brain) is sufficient to satisfy the phenomenological attitude which I have sketched in the introductory part of this paper? Is it sufficient for encountering that which—for Heidegger this is the truth of Being—is given in the work of arts?

A good place to begin to respond to these queries above might be from the attestative experience of artists themselves, or by extension from our own experience of artworks. One such was given by Lellouche, whose account in *Art and Sciences* examined, for instance, the difference between the Montagne Sainte-Victoire painted by Cézanne and a simple photograph of it, and questioned why the former made us dream and the latter does not go beyond a mere portrayal of nature: a mountain. For Lellouche, the latter does not capture the essence of art, which “is not a representation of nature in a beautiful way” (Lellouche, 2013). We are aware that some like Burke or even some versions of the transparency theory of consciousness (the versions that defend realism) dispute this idea^8^. Without commenting on those examples of realism, I argue that if art signifies in a way other than representing nature, then there is a profound difference between “*perception” of art* and *pure perception*, the latter capturing the language of *everydayness*. What Lellouche tried to express, like many other artists and contrary to common belief, is that “perceiving” or contemplating an artwork differs from perceiving the world of everydayness. The studies which Pepperell described above, according to which there is a known strong tendency, in cases of abstract art, to organize indeterminate objects into recognizable and coherent images, should surely make us wary of any “veridical impression of reality,” although this might equally have a biological advantage in terms of survival. If the entire visual system has to undergo this not-easy-process (not excluding that of imposing a semantic meaning) for us to recognize objects, then this would make every perceptive act object-related (it is contingent on *entities*). Also, it would imply that the objects we recognize are not veridically *in that* which we perceive, *in the world*. If this could be said in a restricted sense for the perceptual process, it is most adequate for the perception of works of art. Whereas no one would argue with me that a computer is before me on my desk or that *this* Mount Everest before which I stand in Nepal is the greatest mountain ever seen by any human, they may argue that Paul Cézanne’s painted Montagne Sainte-Victoire provides an *accurate and unequivocal representation* of *this* mountain in Aix-en-Provence of southern France. The two levels of reality are not *there* in the same way. What is the connection between the artworks—the strokes, the styles, the dexterity, the oil and acrylic paints, the balsa wood and the polymer clay of the painter and the sculptor—and reality, the world that is before my eyes? If artists claim that art does not give us a representative picture of everydayness, it is because they know that they express something beyond realism and veridicalism, beyond what might be immediately obvious through *mere perception*. It is this *something beyond* that fascinates all lovers of art when they give themselves away to the works of art, thereby *borrowing the eyes* of the artist.

From the above musings we are tempted to say with some sort of security that artists are practical metaphysicians. The gaze of the painter about the world does not follow a path of naïveté, of credulism, of faith in perception as those ideas that claim that artworks disclose the everydayness of their subjects might surreptitiously imply. I have already cited Burke as an example of this faith in perception in the footnote above when he claims that “the images in painting are exactly similar to those in nature.” Against Burke’s realism, I argue that the painter’s vision distinguishes itself from ours. He does not gaze the way we do and he sees what we do not see, what remains imperceptible to our normal eyes. Richir’s intuition captures what our analysis highlights, in a more profound way:

The lesson, in any case, is that a seeing which does not blink with my gaze is a seeing which sees nothing, or, which amounts to the same thing, a seeing which is forgotten in the seen by merging with it, by losing itself in it—and that is to say, how much *the physical constitution of the optical signal* through our *sensory-motor apparatus is insufficient* (although playing as a necessary condition) to understand here what is happening, where it is the whole *Leiblichkeit* […] that is at stake (Richir, 2006:404, my translation).

The time is not ripe for us to comment on this passage in detail. Suffice it to say, however, in simple language that the biological eyes, in their physical and chemical physiology---that is, our visual nervous system, what Richir called the sensory-motor apparatus---are not sufficient^9^ to understand what is going on in the *Leiblichkeit*, which, for Richir, as we shall see later, is clearly linked to the sort of experience that corresponds to those of artworks. This physical constitution of signal, like other organs too, is captured by the term *Körper* in Richir’s phenomenology. We translate it henceforth as a sort of material body, which is given in object-representation, the very opposite of *Leiblichkeit*, which is associated with a sensuous perception (*voir*) and which is beyond object-representation (Richir, 2006; see also Husserl^10^). We can see that the former, as a spatial exteriority, carries no affectivity with it. In a very condensed phrase this connection is spelt out: “and via *seeing* its *Körperlichkeit* is formed” (French version reads: “*et par le voir*… *se constitue sa Körperlichkeit*”, Richir, 2006:292). The phrase simply relates *Körperlichkeit* with object-perception, where *Körperlichkeit*, a sort of material corporeality, is understood as a *Leibkörper* (the body understood as being composed of the *psychic* and the *physical* or *material* part) stripped of its *Leiblichkeit*.

Two levels of reality are at stake here: To our initial analysis that the material body is not sufficient for the contemplation of artworks, which Richir reinforces, we can add that the material body does not suffice even for the perception of spatial exteriority [‘‘of such and such, that is to say, for there to be a perceptive doxa, the material body (Körper) engendering a space’’^11^ 2006: 278]. The simplest motivation is the assumption that material body as *Körper* cannot perceive material body as *Körper* (the psychic part that is *leiblich* is failing). If the physical constitution of optical signals was insufficient for *mere perception*^12^ and if *mere perceptive vision* is affected by irreality, as I claim in the previous footnote, how could it then at the same time be sufficient for a more than meets the eyes perception as given in the contemplation of works of art? For were the material body to suffice in the contemplation of artworks, then all of us would be one or all of these: Leonardo Da Vinci, Vincent Van Gogh, Pablo Picasso, Rembrandt, Cézanne, Jean-Michel Basquiat, etc. This can be extended to music, poetry, dance, and other performative arts in like manner of declining the sufficiency of all the sense organs since that would lead us only to the same sort of naivety documented above. What then suffices for the contemplation of artworks beyond *things* or *entitie*s? In other words, what must happen to complement the insufficiency of *mere perception*? That is, what would satisfy the phenomenological attitude, which I have mentioned in the introductory part of this paper?

I shall turn to the above question later on. In the meantime, I want to observe that there is a big gap between *seeing a thing* or seeing with the *material body* and *contemplating a work of art*. With this we think we have come very close to the answers to the questions posed initially, according to which it is obvious that the nervous systems are insufficient in satisfying the phenomenological attitude above, which remains our guiding principle in this paper. Besides, the nervous systems do not suffice to lead us to encountering that *something beyond*---which for Heidegger is the truth of Being^13^ —that is given in the works of art. We have to look for an answer elsewhere.

This will become evident below where we return to Richir’s phenomenology, which proposes another form of corporeal “perception” in the “*perceptive” phantasia.* In this form of “perception,” we shall see how the material stuff or objects are neutralized—moving from “stuff” or “matter” (*Ding* in German) to *Sache* (literally it means something) or the “real,” which is a “phenomenon” experienceable as “sense”—since what this “perception” discloses “has never been” and “will never be” (virtual). By becoming a “phenomenon,” the *Sache* can no longer be attributable to the metaphysical condensations of *truth*, *Being*, and *world. These are* inexperienceable. Contrariwise, the *Sache* disclosed in the works of arts are henceforth liveable in experience, involving *Leiblichkeit* as a sensuous opening to the world. In these senses, the first and the second problems would be solved. But before this, in the following section, we shall show that that which is “perceived” in the work of art is no longer an object as they are given in the everydayness of perception but a “transitional object” between “figuration” and “infiguration.” In other terms, it is in transition between “reality” and *the phantasia* but illocalizable in perceptive and imaginative objects. So, it is neither a real object nor *a phantom.* We shall come to explain these.

## “Perception” of the “Sache” in the “*Perceptive” Phantasia*

Without repeating Richir’s whole confrontation with Husserl’s *Phantasia*, *Bildbewusstsein*, *Erinnerung*, in which Richir makes a new discovery, the *phantasia*, I extract from it just the most salient points for us. Without also treating in detail some of its characteristics---that it appears and disappears in the manner of a flashing lightning and in an intermittent and discontinuous way (*blitzhaft*), that it is proteic (*proteusartig*), and mostly not present (*nicht gegenwärtig*)---as they appear in Husserl, suffice it to mention that Richir refused intentionality to *phantasia* which Husserl calls *simple*^14^ (Alexander, 2013) and which the latter has sometimes attributed a *Bildobjet*. For Richir, *this simple*^15^ *phantasia* becomes *phantasia* as such and it is unlike Husserl never to be attributed a *Bildobjekt*. From here, however, intentionality can arise through an architectonic transposition^16^, which transforms pre-intentional objectless *phantasia* into imagination^17^. Imagination on its part is imbued with intentionality. Richir further explains that *phantasia*, because of its proteic nature that makes it non-intuitive, nebulous, and always changing in its interior, cannot represent any intentional object. This is why it is *not positional* (*not intentional*) and therefore could not transmit via the role of a mediation—like that of a “copy” if we think for instance about imitating an artwork by producing a “form” of it—the *position of any object*, whether of a perceptual entity in the world or an imagined object (which may or may not be in the world). The reason is that in *pure perception* and imagination, unlike the *phantasia*, objects are intended through the very act of intentionality. For Schnell this captures the revolutionary character of Richir’s *phantasia* that established a new point of departure in phenomenology, away from the intentionality of Husserlian consciousness (which is always a consciousness of something)—here there is an objectifying act of perception where perception is the model of relating with the objects (Schnell, 2011). This leaves us wondering what the *phantasia* perceives at all since it does not “perceive” intended objects.

Richir evokes Husserl’s ‘‘perceptive’’ *phantasia*^18^ in §18 of Husserliana XXIII (Husserl, 1980) to respond to the question above. The “perception” takes place in the *phantasia* and is of another genre than that of *perception* (and *imagination*) because the “object” is neutralized in it. For Alexander: Richir’s “perceptive” *phantasia* “does not perceive reality and its ‘perception’ is unfulfilled […] without image” (Alexander, 2013, my translation). Thus, if we speak of the neutralization of the ‘‘object’’ in the *phantasia*, it presupposes, in Richir’s eyes, a figuring (‘‘*une figuration*’’) of *something*, though not of the genre of pure perception (as *Wahrnehmung*). But since the object is neutralized, that is, since it is not perceived as an entity or an image, the *phantasia* ‘‘perceives’’ the infigurable (‘‘*l’infigurable*’’); thus, if that which is ‘‘perceived’’ in the ‘‘perceptive’’ *phantasia* is neither an *entity* nor an *image*, it is *in transition*. To this effect, following Winnicott’s *transitional space*^19^, Richir posits that *that something* (we call this *the transitional object* as distinct from the pure object of pure perception) which is figured out in the ‘‘perceptive’’ *phantasia* lies *in transition*, i.e., between the ‘‘figurable’’ and the ‘‘infigurable.’’^20^

There is the ‘‘figurable’’ part in the different spheres of arts. But this ‘‘figurable’’ part is the ‘‘object’’ ‘‘perceived’’ as transitional; it is the perceptive part. In the case of music, the ‘‘transitional object’’ is the sounds emitted by voices or instruments; in a novel, it is a ‘‘Bildsujet^21^” figured in the narrative; in poems, it is the more or less elements figuring the referents of language set out by the words (Richir, 2008). So for Richir, the “transitional object” has its figurable part which lies in the “perceptivity” of the “perceptive” *phantasia*. Following Winnicott, the transitional object lies in the transitional space, a space of free play and pure virtuality (*1bid*, 180). The infigurable part of the transitional object is found in the *phantasia* that is non-object-intended. This allows Richir to claim that the paradoxical connection between the figurable and the infigurable in the “perceptive” *phantasia* is not-intentional like in pure perception or in imagination. The transitional object is unable to connect intentionally to that which it opens up as infigurable; for this reason, it is therefore evident that nothing in this connection is positional or susceptible to ontological veridical identification. If the relationship that holds between the figurable (or “perceptive”) of the transitional object and the unfigurable in the (“perceptive”) *phantasia* is not one of intentionality, does this not leave us perplexed and frustrated in our struggle to understand what is happening there? What are we then to make of the authentic nature of what is perceived there after all?

It is in that direction that Richir assuages our worries. The problem ensues perhaps from our wrong supposition that the ‘‘transitional object’’ is an object of a certain type that is ‘‘perceived’’ in a way. No. It is nothing about perception here for what is perceived is a sort of feigned perception (*Schein-Perzeption*). Thus, if Richir speaks of ‘‘perception,’’ then this does not imply that this or that entity is truly perceived; whatever it is that is ‘‘perceived’’ is perceived in transition but not as a concrete object. To explain the nature of what is perceived here beyond the semblance of it, Richir comes to a novel vocabulary, which characterizes that which is ‘‘perceived’’ in the transitional sphere of the ‘‘perceptive’’ *phantasia*: ‘‘Sache^22^” (2008: 181), derived from its German origin but without adequate French or English translation. It is this “Sache” that prevents the confusion that we were perceiving an object as such, and makes it possible for the figuration (“perceptive”) to be able to provide access to the infigurable (the *phantasia*) that is simultaneously implicated. In simple terms, that which is “perceived” in the work of art is “perceived” as “Sache,” in the “perceptive” *phantasia*, and is astride a figuration and an infiguration, in transition between “reality” and *phantasia*, but illocalizable in perceptive and imaginative objects as such, i.e., not as real objects and not as phantoms. We can obviously notice that the “Sache” lies in a sphere of indetermination, in a space that leaves a taste of insatisfaction. Richir writes that this indeterminate rapport of “figuration” and “infiguration,” in which the “Sache” lies, “is, in each case, that which we encounter in artistic creations and receptions” (2008: 187).

What phenomenological implication does this have for us, especially for our resolute question at the beginning of this investigation: how the *truth of Being* is given to us in the work of arts? I attempt to respond to this in the following section, with the view of showing, in the end, how the metaphysical attitude presupposed in the above phrase could be neutralized in a phenomenological attitude that gives us access to the essence of artworks. This attitude will be concretized in a totally new form of *mimesis* that is neither *specular* (imagination) nor *from without* (perception) but *active*.

## Discussion

### *Mimesis*^23^ of the “Sache” in the *Phantasieleiblichkeit* as Phenomenon

Several consequences can be drawn from the above realization that the essence of art (for Heidegger this is the *truth of Being*) is given neither as an entity that I can identify as this or that, nor as a *phantom* that is a deceptive copy of an unreal this or that, but as a *Sache.* In consequence, the *Sache* is neither a *reality* nor an *irreality*, but a “concreteness” (*concretude*), which is able to grip *something* while being oblivious of the *something’s* “whatness.”

First, as it is palpable that the “Sache” is neither a “reality” nor an “irreality,” we can ask: what is then its status: a *pure possibility* valid for imagination or a *real possibility* as in the case of memory (Richir, 2000)? Far from being a reality (which *is* now), nor a possible reality (which will become), nor a reality which was in time, Richir explains this “Sache” as “purely virtual.” By this he understands that which, comparable to the quantum mechanics, “*without being present*, being linked to the current by bias of the potential, however, does not exercise lesser real effects on the phenomenological field” (Richir, 2008, my translation adapted and italicized). The virtual is like an impossible possibility that shatters the poles of that which is currently present, even though it is itself not currently present. Nevertheless, it is not less real. Contemplating a piece of artwork is not the same as being in reality or in irreality, but it implies perceiving a virtually non-present “Sache” that penetrates a piece of art, whose effect is as real as a veritable present (with this the “first problem” is solved). It is opening that *world*, which, though not here present with me, is nonetheless a *horizon of disclosure* that has a lot to tell me. It is not same as being in the world; rather it is opening a virtual world.

Second, that the “Sache” (we have understood this in Heideggerian terms as “the *truth of Being*”) lies in a sphere of indetermination, mirrors the essential of artworks as lying in its escape of socio-historic and cultural codifications (symbolic or metaphysical determinations, if you like):

‘‘it is that the essence of art is not, in this way, in what comes under socio-historical-cultural analysis---the analyses of codes---but precisely *in what radically escapes from it, in what transgresses them* by modifying them in an unexpected and original way, *in what makes them*, therefore, the *cantilever* (*le porte à faux*) *of phenomenon* as nothing but a phenomenon’’^24^. (Richir, 1991, my translation).

If art speaks to us, it does so through this essence that defies the codes, and if the codes (that is, the regime of credulisms saturating our perception by determining them in advance) are defied, it is precisely because there is something in the codes that leaves the artists with a feeling of dissatisfaction. Whereas the regime of perception is that of entities, i.e., of the present, the regime of art is that of *Sache*, i.e., of the absent that transgresses and defies our natural attitude. It is these elusive, ‘‘dissatisfactory’’ feelings that artworks try to communicate to their receivers: audience, spectators, etc. All of these make the essence of art somewhat elusive, reduced to the barest minimum (via a phenomenological reduction and *epoché*) of the apparent, because instead of a *presentness* that is, we have an *absence* whose presence is felt [the apparent]. Therefore, Richir understands the essence of art, i.e., the ‘‘Sache^25^,’’ as nothing but a phenomenon. This makes a lot of sense if we understand that the phenomenon, otherwise the appearance, is in itself irreducible^26^ to that which is given in presence or identification, be it real (perception) or a copy (imagination: this is another form of perception which we shall not handle here).

It is this aspect of the phenomenality of art that highlights the ‘‘Sache’’ perceived in the works of art as an ‘‘aesthetic moment,’’ and not just a pure metaphysical moment, dissociated from our experience. In his paper from 1991, Richir translated *truth of Being* with *The Truth*^27^ *of Appearance*, thus turning the metaphysical condensation upside down. Here appearance spans the sphere of phenomenality, if we understand that the metaphysical category of *Being* is accessible to us, when it is perceived in the ‘‘perceptive’’ *phantasia* as the *Sache* of an artwork, *only* as a *phenomenon* and is thereafter irreducible to no other thing. This truth can only be truth via appearance, i.e., the phenomenon, which correlatively and simultaneously puts Being into parenthesis. It is in this purview that the phenomenological precondition at the introductory part of this work is realizable, if we understand by that the satisfaction of the precondition for phenomenological *epoché* and reduction, at least in the sense proposed by the founder of phenomenology, Edmund Husserl. Though the latter had demanded in his *Logischen Untersuchungen* for a return to the *Sache* itself, which are nothing but the ‘‘phenomenon,’’ this latter would be impossible without (1) a metaphysical preconditionlessness, i.e., a putting into parenthesis of the metaphysical conditions of ‘‘Being of the World’’ (*Sein*) and ‘‘the totality of entities’’ (*Seiende*)---*epoché*^28^ (HUA III) fulfills this precondition—and (2) the opening of an original transcendental referentiality, which leads only to the sense of Being—phenomenological reduction (HUA XIII) fulfills this precondition (Husserl, 1973: 434; Schnell, 2019). We can now see how Richir’s claim that the phenomenological is required for the aporias of the metaphysical is satisfied, and, in doing so, has opened up the possibility of translating how the metaphysical condensation of *truth of Being* is phenomenologically given in the works of art (the “second” problem is solved). To this must follow, however, the attempt to understand how this truth of Being is given to us in aesthetic experience. How does the artist and the receiver of his work gain access to that something which a particular artwork tells us?

This question leads us to pursue the last, if you like the third, implication of the “Sache” lying in the sphere of indetermination. Since the “Sache” is this unstable and always flashing phenomenon, always on transit between an upsurge and an eclipsing disappearance at the heart of an artwork (Richir, 2002), it then shows the artist’s source of inspiration, that is non-conceptualizable to fly in the wings of Kant’s third critique (Kant, 1987, 2001). For the sake of emphasis, we note again that the indetermination is captured by a sort of emptiness, blankness, restlessness, which through a sort of dissatisfaction vivifies the artist’s power of the mind (*Einbildungskraft* in Kant’s language), i.e., setting the *phantasia* at work. This level of operation is for Richir a process of phenomenalization or of schematization in which, though there is neither an *entity* nor a *non-being* to imitate, an aesthetic sense, a concreteness or *phenomenon* begins to build up in the artists’ minds. Now the artist imitatively perceives this concreteness in a direct and non-mediating way via what Richir characterizes as an *active, non-mirror-like mimesis from within.* Thus, he imitatively perceives the concreteness neither through any *passive* mediation of a *mirror* (not a copy, not a model) nor *from without* (entities) but directly *from within* (*du dedans*) and *active.* “Active” means that the concreteness is not imposed on the artist since it is produced from the sphere of free play, of the transitional space. If it were, then this would be in the form of a mode (mirror-like) where this or that image would be suggested by the imagination. “From within” characterizes the creative activity of the *Leib* (the living body) in its psychic and indeterminate sensibility to the world. If the indeterminate sphere of the *Sache* enlivens the power of the mind into activity, it means that the living body (corps vivant, i.e., the *Leiblichkeit*) too is given life and this process is what gives life and body to a work of art. Thus, Richir writes:

In this way too, there is no work of art without *horizons of indeterminacy*, and without what, by being engulfed in them, *the living body* (*Leib*) of the “receiver” brings to it with its *kinestheses*, not of its real kinesthesis, accompanying the movements of the real material body (*Körper*) but of his kinestheses in *phantasia*, pertaining to a *living body in phantasia*, of a *phantasieleib*, not “connected,” so to speak, to the real material body, and constituting what we usually call psychic, if we want, concrete but immaterial “space” (and “time”) (Richir, 2002: 70, my translation).

What Richir phenomenologically describes is a process of formation of aesthetic sense generated by the indetermination of the phenomenon that the artist is beginning to grasp. It is, so to say, a process of activity (Kinesthesis) taking place in the interior of the *phantasia* that can never be devoid of the living body (*Leib*). To contemplate a work of art is to experience this indetermination or restlessness that the artist exploits; but for Richir this is unthinkable were the living body not to animate the *phantasia* (*phantasieleib*), all of which for him is a psychic activity. It then turns out to have a very important consequence for the receiver of artwork, i.e., the artist and the contemplator or spectator. For an artwork to be worth the name, the receiver has to feel (via empathy or Einfühlung), in the *phantasieleib*, this restlessness in a direct, active and non-specular manner (for Richir the meaning of *mimesis, active et du dedans, non-speculaire*). A detailed passage from Richir’s *Art et Artefact* highlights this very subtle point:

By doing so, finally, the artist goes through his real body (by putting it into action)—but just what is necessary so that the body of *phantasia* comes to play (the *phantasieleib* put into, extremely fleeting and mobile play)—and through the indeterminacies of its (body) infigurability, schematizations without concept and kinestheses in *phantasia* of this body in *phantasia* which all play out by being *felt* there but not being figured, in the hollow or at a distance from figurations. It is this body of *phantasia* which, radically unrepresentable and infigurable, comes to represent itself in a certain way, all indirect, despite everything, but *as the very pulsation of life in the work of art as a phenomenon* […] But every real artist *exposes* to others, in a sort of immediately vulnerable absolute abandonment, something of his *Leiblichkeit*, however, infigurable in itself. This is why these always precarious and to be resumed “exhibitions,” are in principle always in search of an *impossible accomplishment.* (Richir, 2002:71, my translation).

Before I comment on this passage, let me emphasize that Richir is not talking about the role of the artist as such. He is only highlighting how the artist “perceives” and how this “perception,” if it is successful, is later passed on to the contemplator of artworks. In simple terms it is the living body’s mobilization of the indeterminacies of the *phantasia* which is felt by the artist that is *responsible* for any artistic perceptive experience and that is *felt* by an artist in a work of art. Thus, the artist impresses this indetermination of his *phantasieleib* (the living body of his *phantasia*) in his artwork and which is hoped to be passed to the receiver. While contemplating a work of art, the work would be worth its name, its truth of Being would have been disclosed, if the viewer could, via an *active, non-specular mimesis from without*, affectively “perceive” in his “perceptive” *phantasia* that “Sache” which left the artist restless and which his living body’s *phantasia* (*Phantasieleiblichkeit*) has impressed on the artwork.

We are now going to concretize the above intuitions of Richir in a particular form of art: Theater.

#### In the Theater

The theater constitutes for Richir a paradox owing to the immense task required by the actor or comedian. Though in a theater two sets of parties are present in real time and space with specific bodies (*Leibkörper*), the actors and the spectators, yet that which is given in reality has to be bypassed in what constitutes a sort of fiction or intrigue. It is here that the immense task of the actor and the paradox of the theater are constituted: talented actors are able to erase themselves and embody their characters, for instance *Okonkwo* in *Things Fall Apart*. This is achieved when the actor, despite his material body (*Körper*) and its kinesthesis, literally lends his living body (*Leib*) to the living body of the character he incarnates, to effectuate his elision, though neither through any figuration in perception nor in imagination. Hence, though the actor appears on the stages of the theater, it is the character who is nevertheless “in presence… without ever being *present*” (Richir, 2003). This is a way of saying that the character is in his “presence” there, though absent in a real spatio-temporal sense. Now if the character *Okonkwo*, whom a specific actor, say Denzel Washington, embodies is neither present in a real (object of figuration in perception) nor in an imaginary (image or object of figuration in imagination) spatio-temporal sense, how is he then there in his “presence”? I cite a passage to show how Richir would respond to this question:

In this way, since the character, if he is well “embodied” by the actor, is not *present* in an *intentional act of perception or imagination*, he is *in presence*, as in himself intuitively infigurable, *in phantasia*. And if he *is there, in the presence*, but *not present*—and since it takes the actor to “incarnate”—he is nevertheless *the object of a “perception,”* in another sense of these two terms in phenomenological quotation marks… in what Husserl aptly calls a “perceptive” *phantasia*. (Richir, 2003).

The modus of presence of the character in the spatio-temporal world of the theater is therefore virtual, since this presence is of a reality that “never was” and “will never be” in an intentional act of perception, unless *transposed* into imagination, but in the transitional space of the “perceptive” *phantasia*, where though the character is “perceived,” is nevertheless not in the manner of an object. Far from it, the presence that is *there* is that of a “consistence” or of a “concreteness” (*Sachlichkeit*) of the character to which the “perceptive” *phantasia* opens. Now the character floats in the transitional space, following Donald Winnicott, between reality and phantasia. Here its reality as “object” is neutralized.

It requires an enormous task of talented diligence on the part of the actor to achieve all these: to be able to embody the character via self-elision and thereby neutralize the character’s real spatio-temporality. To achieve this he needs, not to imitate specularly the imaginary representation that otherwise would be the character but to *feel* via empathy (Einfühlung) the character who never existed nor will ever exist in reality, outside the theater, thereby effectuating an *active and non-specular mimesis from without*. In doing so, in the depth of *Phantasieleiblichkeit*—which in itself makes possible the affectivity—he gains access to the character’s interiority: his emotions, affectivity, mimics, gesticulations, sense, and sensitivity, which now, in their own turn, perform the wonders of the theater. If the actor is diligent and succeeds not to slip off, transposing this interior access to the character into a mirror-like image, in which, for instance, he (say Denzel Washington) narcissistically projects the structure of his personal phantasm in his character (Okonkwo) and would thereby lead the spectator to imagine the character that is being mediated, then he is able to perform the second part of the task. This consists in transposing the spectators into the virtual spatio-temporal theater, where the character is truly and really alive, so they could “perceive” the character in their respective “perceptive” *phantasia*, when their living body mobilizes their *phantasiai* into activity. The spectators succeed on their part, when they are then able to “perceive” (*feel*) the character (Alexander, 2013), in this case Okonkwo—and not Denzel Washington—as real, *hinc et nunc*, although no Okonkwo had ever existed or would ever exist, except in the *Things Fall Apart.*

## Conclusion

Our presentation has tried, among other things, to outline how Heidegger and Richir varied in their conception of art. Though they varied as to their perspectives (Heidegger followed a strong metaphysical/ontological approach to art, whereas Richir showed how the metaphysical could be experienceable via a phenomenological approach), they agree that there is something which the works of art want to tell us: For Heidegger it discloses the truth of Being and for Richir we come to experience this truth of Being not as an abstract universal but as concreteness—“Sache”—phenomenologically, corporeally, through a special type of imitative “perception” in the “perceptive” *phantasia*: *non-specular mimesis*, *from within*. If the world which Van Gogh’s shoe painting discloses to us is that of a peasant farmer’s everydayness (in its ontological sense), and if artists, interpreters, and contemplators of his works are able to “perceive” (feel) that which the work is trying to tell them affectively and corporeally, then we hold that there must be something unique to all artworks, and that participation must be a unique way of entering into the essence of arts, the “Sache” they disclose. But in participating, it has to be quickly added, contemplators of artworks do not, for us, do so in the sense proposed by Heidegger, as though there was a world that existed or a world that will exist.

If we participate in that which artwork desires to tell us, we have to say that, following Heidegger, it is because artworks are *always open opportunities* to be in the world in a way that destabilizes our *normal modus of being in the world*^29^. This last point needs a little clarification. From our earliest cradle in the world, a given “order” named civilization has always sought to tame our natural instincts and cut them to size. We learned to give certain labels to given impressions of our perception. The world that dictates for us what things are and are meant to be judges for us. Our time and spaces were inundated with events that matter less to us and speak less to our personal lives. The most frustrating aspect of this way of being in the world is the alienating effect that imposes norms and restricts subjects from encountering things as they appear to them. As we can see, in such a world nothing can be credited to the subject.

Against this model, art offers us another mode of being in the world by having a say in it: here we possess the possibility to be ourselves, a possibility which belongs to subjects in the world as such and which can be exercised without the censure imposed from outside; for here too subjects can “judge” the world—though without using concepts—and not be judged. Things are perceived beyond mere labels. Time and space no longer conform again to the order of objectivity and normativity. Subjects become so to say *anarchic*—that is to say without *arche* and a guiding principles. Art feels those gaps and wants to fill them in. Just for the sake of it or just for the pleasure attached to be anarchic. It is thus not surprising that subjects find pleasure in doing art: it gives them wings to fly. We can now see that art offers humans something that is basic and an essential component of human life, but which right from the earliest cradle is denied them: liberty. When we contemplate art, we escape alienation and therefore participate in ordering the world in our own terms and creatively contributing to articulating the infinite indeterminacies in it. We escape the ruthless estrangements of being in the world.

Part of this liberty, more than a mere perception of a “Sache,” is the capacity to bond with the “Sache” itself in a way that neutralizes one’s material body. Here not only does the object disappear, but the subject too vanishes into a timeless absence, where the flow of time is, so to say, relativized. Contemplating Van Gogh’s shoe-painting, I enter into a world that is not just known to artists, some experts, and interpreters of the work of art, but that is also available to me, *hic et nunc*, with its real absence. Concentrating only on the metaphysical and ontological aspect of artworks, Heidegger was not able to articulate what the subject^30^ contemplating art brings with him. The consequence is that *the truth of Being, the world* of this or that particular artwork has always been disclosed, and always in advance. If Richir made a contributive corrective to this manner of contemplating art, then this contribution is rooted in the subject’s active participation. And it is only such participation in which the gap between subject and object disappears that can do justice to Richir’s idea of *active non-specular mimesis from within.* It is also this qualitative aspect of what the subject brings with him or her that is rarely thematized in some theories of perception, e.g., in neuroscience, where *the necessary* but *insufficient* quantitative measure of brain activity is the focus. Perhaps these theories might need to take these qualitative aspects of perception into consideration.

## Author Contributions

DE is the sole author of this article and is responsible for its content.

## Conflict of Interest

The author declares that the research was conducted in the absence of any commercial or financial relationships that could be construed as a potential conflict of interest.

## Footnotes

1 In *Being and Time*, this connection is evident too. Heidegger had written at the very beginning line of §44: “From time immemorial, philosophy has associated truth and Being” (Heidegger, 1962/2001); and it appears that Heidegger is applying it here again. Accordingly, *Truth* is here a mode of *Being* for Dasein.

2 Obviously, Heidegger is endorsing Hegel’s thesis—appearing in the *Vorlesung über die Ästhetik* here—according to which great art was the highest way in which truth provided itself existence. It is true that Hegel later held that this great art, from the point of view of its greatest determination, lies in the past. Following this, Heidegger’s interest was to see if great art remained the way in which truth happens for our historical Dasein.

3 This approach is radicalized in a sort of subjectivism that operates according to the laws of subject–object dichotomy. This tendency of seeing and consuming works of arts can be found in a passage of Heidegger’s *Questions concerning Technology and other essays*: “A third equally essential phenomenon of the modern period lies in the event of art’s moving into the purview of aesthetics. That means that artwork becomes the object of mere subjective experience, and that consequently art is considered to be an expression of human life” (Heidegger, 1977:116). This tendency reduces works of arts to simple objects like any other through which a subject can derive a meaningful experience. To derive this aesthetic—and we should not forget the Greek root of this which can be translated in terms of sensation, sensuality, feelings, etc.—experience, the subject needs first of all to leave his own most subjective domain to relish the artwork after which he would satisfactorily come back to the abandoned domain, as though he had acquired something outside himself which he never had before. Contrary to this movement, and that is the point Heidegger subtly tried to establish in *The Origin of the Work of Art*, works of art have a profound place in our everydayness, re-echoing the thesis of *Being and Time* where Dasein’s ontological constitution as b*eing-in-the-World* was paramount.

4 This is, however, not to imply that *Being* cannot be given to Dasein in a context of encounter. Contrariwise, Plato advocated for this subjective encounter when he wrote: “Now, as we have said, every human soul has, by reason of her nature, had contemplation of *true Being*” even when it is obvious that the italicized is in reference to the world of forms.

5 In the history of the philosophy of art there have been varied ways of experiencing that which artworks disclose. Some of these are imagination, *mimesis*, and perception. In this paper I examine only perception.

6 For some like Richir, the other senses are drawn up to the height of the abyss opened by the most *singular* of all the senses: vision. That is to say that they can converge around vision. I can see that whose sound I hear, see that which tastes so and so or which I touch or that smells rotten or fresh. However, the singularity of sight lies namely, contrary to all the senses that could be perceptible either for themselves or for another, in the fact that it cannot perceive itself. But later, this metaphysical tradition that privileges sight would be overturned by Merleau-Ponty for the first time in the history of thought. In place of sight, the tactile organ of body sensation in the chiasm of “touching/touched” would be instituted (Merleau-Ponty, 1964a,b).

7 Grossberg and Zajac (2017) have described paintings of some illustrious artist in this light. Some of the models explored were the most advanced of all computational theories and they include the Form-And-Color-And-Depth (FAÇADE) model of 3D Vision, figure-ground perception, and the 3D LAMINART model. These models are able to explain how the brain sees “what happens in a viewer’s brain when having a conscious visual experience, including the viewing of a painting.”

8 To give just one example, let us quote Edmund Burke, who can be ascribed a kind of realism: “But painting […] can only affect simply by the images it presents […] *because the images in painting are exactly similar to those in nature*” (Burke, 1990). Burke is not alone in this context. In the analytical philosophy of mind, too, a similar idea is represented in the theory of the transparency of consciousness. We refrain from details to this regard.

9 This does not preclude the fact that the eyes provide the necessary preconditions for perception. However, these conditions do not necessarily satisfy their sufficiency for the experience of painting, for instance. Were the eyes sufficient, perhaps the knowledge or the acquisition of the knowledge of physics might be satisfactory for being a painter, where the studies of the physical laws and nature of color might do. This would only lead into absurdity (Richir, 1993). In that sense perception would be in fact nothing but the registering of the signals corresponding to the physical laws of nature. To experience blue would mean nothing but perceptively registering and recognizing the signs indicated by blue. But what artists do goes beyond registration of physical signs mediated by the eyes. This can by way of analogy be extended to the experience of music, which goes in a way beyond the mere perception of the physical signs, for example of a particular note on the piano, via the olfactory system. For what the musician composes cannot be understood as renderable in mere “perception of successive sounds” (Richir, 2005). Summarily we can agree on this: the *material body* (eyes, brain, etc.) is necessary for perception of *material bodies:* colors and sounds, etc. However, it is another thing for it to be sufficient for contemplating the essence of artworks.

10 Richir bases his analysis on a reading of Husserl’s *Idee 11*, especially where Husserl wrote that purely “visual subject” could not have any appearing *Leib* (148–150). This marks the radical difference and opposition of Leib and Körper. Whereas the latter corresponds more to “vision” or “seeing,” i.e., to object perception, the former cannot be localized in vision (Richir, 2006).

11 The French version reads: “…*de choses telles et telles, c’est à dire pour qu’il y ait doxa perceptive, le “corps*” (Körper) faisant espace,” p. 278.

12 It might help to add that, for Richir, vision is localizable neither in the eyes nor in the object. Either one might say it has neither organ nor its organs are insensitive or one can say that vision is everywhere and no-where (Richir, 1975:100, 104). Not only are there unknown sites for vision in the eyes, it is also affected by a sort of irreality. That which is given in vision does not follow the order of the real: “the appearance which is phenomenalized in it is unreal, of an “order” quite different from the other sensations: it is of the order of phantasmata, of “pure” appearance, of image or simulacra, floating everywhere and nowhere, without original anchoring in the flesh, be it the sensory flesh or the peripheral cosmos of sensations; or rather it is unable to anchor or locate itself, to acquire “reality” only insofar as there is a convergence […] between a non-visual sensation and itself, for instance between the tactile and visual organ; so that we have a visual tactile.” (French version: “*l’apparence qui se phénoménalise en elle est irrélle, d’un “ordre” tout autre que les autres sensations: elle est de l’ordre du phantasmata, de l’apparence “pure,” de l’Image ou du simulacra, flottant partout et nulle part, sans ancrage originaire dans la chaire, qu’elle soit la chair sensitive ou le cosmos périphérique des sensations; ou plutôt elle n’arrive à s’ancrer ou à se localiser, à acquerir de la “réalité” que dans la mesure où s’effectue un recroisement […] entre une sensation non-visuelle et elle-même”* (Richir, 1975:102). In the end, we have a double character for vision: non-localizable in the body and in the object; it is characterized by the unreal. This last confirms in a way the intuitions of artists, according to which artworks are non-veridical representations of the world. We might question, if *mere perception* does not represent the real, how then can art represent just what the eyes sees—inks, figures, shapes, objects, etc.—of the real world?

13 Though Merleau-Ponty does not subscribe to the physicalization of aesthetic experience, he believed that “vision is the encounter… of all the aspects of Being.” (Toadvine and Lawlor, 2007). In other words, there is an intimate connection between Metaphysics and Phenomenology. If I mention Merleau-Ponty in a phrase that is obviously related to Heidegger, it is because I think the connection is common among philosophers. It is this intimate connection that I explore in this paper.

14 Husserl does not always remain consistent as regards the status of the *Phantasia.* Sometimes he could not distinguish them from Imagination. Sometimes the *phantasia* is named simple (*schlicht*) and possess an object (Bildobjekt). Other times the *phantasia* just mediate figurations. Sometimes they are pure or simple, and sometimes they are not. Richir categorically refuses those *Phantasiai* object (*Bildobjekt*) and intentionality which Husserl has characterized as simple (schlicht). For him, this is *Phantasia* as such, without objects and without intentionality. In this way, he is able to distinguish it from Imagination, which, for him is imbued with intentional objects (Gondek and Tengelyi, 2011).

15 In §18 of Husserliana XXIII, we read, for instance, of what Husserl called “richly passive *phantasia*” (*reichlich, passive Phantasien*). This can be understood as the simple *phantasia*, which in another situation is pure and without object. For Richir, it is not yet activated through a transposition into intentional imagination (Ekweariri, in press).

16 By architectonic transposition, Richir means the movement from one register to another. An example might be the movement from the formation of meaning to symbolic language or a language system, or, as the above example marks, from a positionless *phantasia* to an objectification in intentional imagination.

17 Richir holds that since some of the characteristics of *phantasia*—take, for instance, its intermittent and discontinuous appearance and disappearance—also (Here Richir explores the subtle kinship between phantasia and imagination. A further confirmation of this affiliation between the two is the phenomenon of dream, which, for Richir, is nothing other than a mixture of obscure and nebulous *phantasiai*, in their original phenomenological status, and imaginations, where I recognize this or that place, personality, etc.) appear in the intuitive representation of imagination, this confirms his claim that the *phantasia* is the interior phenomenological base from which the act of imagination is instituted. This involves, as we have hinted above, a transposition from the interior non-intentional and objectless grasp of the *phantasia* to the object-intentionally laden act of imagination with the consequence of an original distortion of the apperception of the *phantasia* to *simulacra* (perceptive appearance). This means that the tendency of the imagination to fix the instability of the *phantasia* on this or that “image” (on this or that model) can lead to a radical destruction of the vivacity of the latter, i.e., to an impoverishment of the most important “activity” (Richir, 2003) of our human mind.

18 This concept was inspired by Husserl’s n. 18 of the HUA XXIII (and in the context of theatrical performance), in which the “perceptive” *phantasia* was treated as a “perception” that goes beyond what is given in pure perception (*Wahrnehmung*). Henceforth, we understand the “perceptive” *phantasia* as a special type of *phantasia*, which is capable of “perceiving” in experience sensations and forms of the real (Richir, 2004).

19 We note that this transitional space is neither object nor subject but a space between the two (Winnicott, 2005).

20 To this regard, we can summarize that in the “perceptive” *phantasia*, two grades of reality are at play: on the one hand, the perception of a figurable, and on the other hand, the perception of the infigurable.

21 *Bildsujet* is the object intended by the image (*Bildobjet*). In the case of the theater, the *Bildsujet* represents the character of a novel who is being acted out by an actor/actress.

22 *Sache* can be further distinguished from *Ding* which represents matter in its prosaic form as entity, e.g., sand, iron, wool, etc. Sache means “thing” without telling us “what” stuff it is made of, wool or iron. It is for Richir a sort of concreteness (Richir, 2004, 2008).

23 By this we understand a special kind of imitative and affective perception. It will become evident in the later part of this section. In the *Poetics*, however, Aristotle treated it as a form of imitation played out in the tragedies, comedies, epic, music, *etc. mimesis* often compares “copies” or “likeness” to their originals. Some like Stephen Halliwell prefer to understand *mimesis* not in the above term but as a sort of signification (Halliwell, 1990).

24 In the original French: “*c’est que l’essentiel de l’art n’est pas, de la sorte, dans ce qui releve de l’analyze socio-historico-culturelle – de l’analyze des codes –, mais précisément dans ce qui y échappe radicalement, dans ce qui les transgresse en les modifiant de manière imprévue et originale, dans ce qui en fait, donc, le porte à faux du phénomène comme rien que phénomène”* (Richir, 1991: 235).

25 We do not imply by this that the *Sache* applies only in art. It is present in any language phenomenon, that is the sphere of “inchoate and indeterminate sense” that Richir terms *le sens se faisant* (sense making itself). For Forestier, this dimension of sense is characterized in Richir by reflexivity between a disappearance and an aiming emergence (Forestier, 2015). If we translate *sens* as “sense,” rather than “meaning,” it is to preserve the inchoativity of sense at this stage. It is to preserve sense from a metaphysical determination which would have exhausted it in advance. Consider, for instance, the translation of *sens* as “meaning.” This latter aspect would imply that there is an entity in the world that is captured by this or that symbolic language. The English word “blue” would therefore refer to a specific type of color in the world.

26 Richir writes: “If appearances have resisted with so many tricks (appearances) to the efforts to reduce them from classical thought, […] it is because there is always in appearances, or in phenomena, something irreducible, which defies the powers of Reason (of non-contradictory identification) […] If this is so, it is because appearances have this paradoxical status of appearing without ever being able to be reduced themselves, until at the end, to such and such appearance where the appearance would have been absorbed, so to speak, and thereby would have ceased to “parasitize” the order of being-true by defying, by an almost diabolical ambivalence or multivalence, the powers of identification” (Richir, 1991: 230).

27 This is not to be understood in the classical sense as they are found in Philosophy since Parmenides (until Heidegger), according to which truth marked a sort of correspondence with Being, i.e., the truth which Richir envisages is that which strives to go beyond its metaphysical status. Such a truth is impossible outside the ambience of appearance and phenomenality. Thus, it appears that the truly true is the appearing, unidentifiable as this or as that, and not reducible to anything. The appearing is the most basic form of the truth. It is therefore irreducible to any other thing.

28 Husserl (1976) and Schnell (2019).

29 Elsewhere we have tried to complement Heidegger’s being in the world with the world being in us; but we think that too is most applicable to the “perceptive” experience of art.

30 In the introductory part of this work, I have already stated that Heidegger was interested in an ontological rather than a subjective approach to art. The ontological approach also explains why Heidegger understood Nietzsche’s “will to power” as “the will of subjectivity,” which “does not allow Being to be,” “what it is,” but rather forces Being into the position of an opponent,” an “object” (Plumpe, 1993). According to Gerhard Plumpe, this accounts for the reason why Heidegger distanced himself from Nietzsche’s understanding of art since it presupposes subjectivity, among other things.
